# The Erie County Hospital

**Published:** 1895-09

**Authors:** 


**A Monthly Review of Medicine and Surgery.**

*EDITORS:*

THOMAS LOTHROP, M. D. - - WM. WARREN POTTER, M. D.

All communications, whether of a literary or business nature, should be addressed to the managing editor: 284 FRANKLIN STREET, BUFFALO, N. Y.

VOL. XXXV. SEPTEMBER, 1895. No. 2.

### THE ERIE COUNTY HOSPITAL.

THE evolution of the Erie County Hospital of today from the late County Asylum for the Insane is fraught with interest to the medical profession, who confidently look forward to the time when its present manifest imperfections and crudities shall have given way to the smooth and systematic working of a well and providently managed charity. And, at this early period of growth, it is certainly encouraging to remember the fact that the widely known and efficient Bellevue Hospital, of New York City, likewise began its career in the abandoned wards of the old insane asylum, which latter building still forms the nucleus of that institution.

It was our recent privilege to listen to Dr. Stephen Smith whose connection with Bellevue dates from its inception, and in his account of the long and arduous struggles of the medical men to free the institution from political control and put it on an independent footing, was faithfully mirrored the inevitable contest which confronts the medical staff of the Erie County Hospital.

The recent conflict between the executive committee of the medical staff and the committee of supervisors, which was so amply exploited by the daily press, gave the public an inkling of the true state of affairs which can but result in good. For months there had been a lack of harmony between the medical and political forces, the latter seemingly being animated by a desire to oppose and obstruct progress instead of coöperating for the good of the hospital. While the duties of committee, keeper, medical staff and superintendent were not yet fully and explicitly defined, proper pains had not been taken by the supervisors and keeper to render themselves familiar with the existing rules and regulations ; and where the animus of discontent and contention was present, discord would easily ensue.

The need of improvement in the matter of diet, service, general cleanliness and sanitation, as well as the proper authority to secure it, was glaringly apparent. Appeals to one authority were referred to a second or third, each on the ground that in complying with the suggestions they would be exceeding their proper sphere of duty or responsibility, and thus were inaugurated and maintained soul-wearying delays and crying abuses. It should be remembered, in attempting to judge the respective merit of the parties involved, that the positions of the staff and the political authorities were and are markedly different. On the one hand, we have a body of educated, public-spirited citizens who have proffered their services freely in the cause of the sick poor. They not only receive no pay but expend greater or smaller sums in going to and fro, in supplying deficiencies in the way of instruments and the like, particularly in the past, to say nothing of the direct and ofttimes large loss of professional income inseparable from attendance at an institution so remotely situated. While they do not desire, we assume, to pose as martyrs or to deny the voluntary assumption of those duties, they may still claim that by virtue of such assumption, which redounds to the advantage of the county and of the sick poor, they are entitled to proper recognition and cordial support.

Under the old condition of affairs, diseases were rarely diagnosticated, much less properly treated, and patients whom a prompt and intelligent handling would soon have restored to usefulness and self-support, were unnecessarily left for weeks or months to be fed and lodged at the public expense, to the exclusion of others equally deserving of assistance. The paramount need of these staff physicians in their altruistic labors is the aid and encouragement of a board of managers which shall be strong, effective and non-political, also a hospital superintendent in whom shall combine the qualities of skill, executive ability and force.

In supplying these wants it is evident to every right-minded citizen that politics should have no voice. Politics and hospitals should be absolutely divorced; they have nothing really in common. To commit the sick poor to the care of politicians who are only too often manifestly indifferent to their duties, or avowedly active in securing for themselves "what there is in it," is little short of a crime against the community and should be frowned down upon by every thoughtful citizen. The remedy for this state of affairs is apparent and should be resorted to at the earliest possible moment. Almshouse and hospital should be absolutely divorced by legislative enactment, and the latter placed under the immediate control of a competent and carefully selected board of managers, who should coöperate with the medical experts of the staff and serve without pay ; then, there being no axes to grind, economy and progress would go hand in hand and in its capacity and good management the Erie County Hospital would soon compare favorably, we believe, with any of the similar great, free institutions of the land.

While the reformations and changes which have been effected during the past two years have been so many and important as to deserve our heartiest congratulations, want of space forbids their enumeration, but they are such as justify the hope that, should the managerial changes above suggested be carried into effect, the good work will go on and in the near future we may be enabled to look with pride upon this, our greatest county charity.

